# Angiotensin II Type 1 Receptor-Mediated Electrical Remodeling in Mouse Cardiac Myocytes

**DOI:** 10.1371/journal.pone.0138711

**Published:** 2015-10-02

**Authors:** Jeremy Kim, Junyuan Gao, Ira S. Cohen, Richard T. Mathias

**Affiliations:** Department of Physiology & Biophysics, State University of New York at Stony Brook, Stony Brook, New York, United States of America; University of Milan, ITALY

## Abstract

We recently characterized an autocrine renin angiotensin system (RAS) in canine heart. Activation of Angiotensin II Type 1 Receptors (AT_1_Rs) induced electrical remodeling, including inhibition of the transient outward potassium current I_to_, prolongation of the action potential (AP), increased calcium entry and increased contractility. Electrical properties of the mouse heart are very different from those of dog heart, but if a similar system existed in mouse, it could be uniquely studied through genetic manipulations. To investigate the presence of a RAS in mouse, we measured APs and I_to_ in isolated myocytes. Application of angiotensin II (A2) for 2 or more hours reduced I_to_ magnitude, without affecting voltage dependence, and prolonged APs in a dose-dependent manner. Based on dose-inhibition curves, the fast and slow components of I_to_ (I_to,fast_ and I_K,slow_) appeared to be coherently regulated by [A2], with 50% inhibition at an A2 concentration of about 400 nM. This very high K_0.5_ is inconsistent with systemic A2 effects, but is consistent with an autocrine RAS in mouse heart. Pre-application of the microtubule destabilizing agent colchicine eliminated A2 effects on I_to_ and AP duration, suggesting these effects depend on intracellular trafficking. Application of the biased agonist SII ([Sar^1^-Ile^4^-Ile^8^]A2), which stimulates receptor internalization without G protein activation, caused I_to_ reduction and AP prolongation similar to A2-induced changes. These data demonstrate AT_1_R mediated regulation of I_to_ in mouse heart. Moreover, all measured properties parallel those measured in dog heart, suggesting an autocrine RAS may be a fundamental feedback system that is present across species.

## Introduction

A transmural gradient in I_to_ amplitude was first detected in canine ventricle by Litovsky & Antzelevitch, [1], and has subsequently been extensively studied (reviewed in [2]). More recently, it was shown that the gradient depended on A2-mediated AT_1_R activation [3,4], which was highest in the canine endocardium and lowest in the epicardium. These data suggested the presence of an autocrine renin angiotensin system (RAS) in the canine myocytes, however the studies were done by whole cell patch clamp of isolated myocytes, so the initial puzzle was: How can A2 effects persist when cells are isolated in a perfused chamber? Gao et al. [4] reasoned that continuous secretion into a diffusionally restricted compartment might be the answer. The only known compartment in myocytes was the transverse tubular system (T-System). They therefore examined the effect of detubulation, and found all AT_1_R-mediated effects were eliminated in detubulated myocytes. Thus an autocrine RAS was a feasible hypothesis to explain the electrophysiological data, and they went on to present a number of hypotheses on mechanism and purpose of the transmural gradient in AT_1_R activation.

The AT_1_R is a G-protein coupled seven-pass transmembrane receptor that is typically activated by the binding of its primary agonist A2. A2 is classically known to exist as a pressure regulating neuro hormone in vasculature, but an increasing number of reports have demonstrated local production and secretion of A2 in cardiac cells [4–6] and autocrine A2 secretion in response to strain [7].

Activation of AT_1_Rs results in 2 sequential phases of receptor signaling. Initially G_q_ and G_i_ are activated. They respectively activate protein kinase C (PKC), and inhibit protein kinase A (PKA) and phosphoinositide 3 kinase (PI3K). One of the major changes that result from G_q_ protein activation is the development of cardiac hypertrophy [8, 9]. In the second stage of AT_1_R signaling, G protein activation ceases, the receptors associate with β-arrestins and the complex is internalized to form a new signal transduction scaffold [10]. AT_1_Rs having point mutations that effectively block G protein signaling have been used to show that AT_1_R internalization can be retained with normal kinetics independent of G protein activation [11,12]. More recently, wild type AT_1_Rs were stimulated using a “biased agonist”—an analog of A2 with substituted side-chains—that selectively activated β-arrestin2 dependent signals and receptor internalization without G protein stimulation [13,14].

Numerous reports have shown that activation of the AT_1_R is involved in both electrical [3, 4, 15–20] and structural [21,22] remodeling of the heart, but these outcomes may be regulated by different pathways downstream of AT_1_R activation. Cardiac hypertrophy typically involves heightened levels of G_q_ protein activity whereas β-arrestin2 activity supports proliferation of cardiac myocytes and does not induce hypertrophic growth [23]. Association of AT_1_Rs with β-arrestin2 mediates positive inotropic effects of the AT_1_R independent of G proteins [24]. This increase in contractility may involve electrical remodeling.

In canine ventricular myocytes, I_to_ α subunit protein (Kv4.3) as well as the α1 subunit of the Na/K ATPase were shown to co-immuno precipitate with the AT_1_R, and both transporters were inhibited by AT_1_R activation [4, 25]. Moreover, Doronin et al. [25] showed *in vitro* co-internalization of Kv4.3 with the AT_1_R. These observations suggested coherent regulation of I_to_ and the Na/K pump across the ventricular wall in canine myocytes was through internalization of the transporters with the AT_1_Rs [4]. Moreover, Gao et al. [4] hypothesized the autocrine RAS in canine ventricular myocytes was activated by strain, which is highest in the endocardium [26, 27]. Activation led to electrical remodeling that increased contractility, which is highest in the endocardium [28]. These effects appeared to be exerted through the second (internalization) phase of AT_1_R signaling, but there was no evidence that G protein signaling was not involved.

In the mouse heart, the ventricular wall is too thin to generate the large transmural gradient in strain present in dog. Nevertheless, if the above ideas generalize to other species, there should be an autocrine RAS that is activated by increases in strain, and its activation should cause electrical remodeling that increases contractility. In the current study, we looked for the presence of an autocrine RAS in mouse ventricular myocytes using whole cell patch clamp studies of the transient outward potassium currents. We also tested the hypotheses that AT_1_R internalization caused inhibition of these currents by inducing internalization of their protein.

## Materials and Methods

### Isolation of Left Ventricular Myocytes

All experiments were done using acutely isolated myocytes from the left ventricles of the mouse heart. The State University of New York at Stony Brook's Institutional Animal Care and Use Committee (IACUC) approved this research. The approved IACUC number on this research is 209215. All animal work was conducted according to relevant national and international guidelines. Wild type mice (7 to 16 weeks old) were euthanized via CO_2_ inhalation in an enclosed chamber. The heart was gently excised and immediately transferred into a 35 mm dish filled with ice-cold Normal Tyrode Solution containing (in mM) 137.7 NaCl, 2.3 NaOH, 5.4 KCl, 1 MgCl_2_, 10 Glucose and 5 HEPES (pH adjusted to 7.4 with NaOH). To cannulate the aorta, the tip of a non-beveled needle was inserted in the aorta and tied together with a 5–0 gauge nylon suture. The heart was then connected to a temperature-controlled Langendorf perfusion apparatus that allowed solution switching. The heart was first perfused with Normal Tyrode Solution at 37°C for 2 to 3 minutes to wash out residual blood in the coronary vessels. Perfusion was then switched to a Normal Tyrode Solution containing 0.16 mg/ml Liberase TH (Roche Applied Science, Inc.) for 9 to 10 minutes or until the perfusion speed dramatically increased, which indicated sufficient tissue digestion. The cannula with heart attached was then removed and manually perfused with KB solution containing (in mM) 83 KCl, 30 K_2_HPO_4_, 5 MgSO_4_, 5 Na-Pyruvic Acid, 5 β-OH-Butyric Acid, 5 Creatine, 20 Taurine, 10 Glucose, 0.5 EGTA and 5 HEPES (pH adjusted to 7.2 with HCl). The heart was placed in a dish containing KB solution and the left ventricle was separated from the rest of the heart. The left ventricle was gently teased apart by mechanical agitation and filtered through a nylon mesh to collect the cell suspension. The isolated myocytes were stored in KB solution at 22°C for up to 12 hours while electrophysiological recordings were being obtained.

### Cell Preparation

For each set of experiments, cells were acutely isolated in the morning and the suspension was divided into Control and Experimental groups for comparison. To insure that angiotensin II (A2) effects were complete, cells in the Experimental groups were incubated for at least 2 hours with A2 (Sigma-Aldrich Inc.) prior to beginning patch clamp studies of either Experimental or Control cells. Experimental cells were subjected to concentrations of A2 ranging from 50 nM to 10 μM or with 5 μM [Sar^1^-Ile^4^-Ile^8^]A2 (SII) (Cleveland Clinic Core Facility Cleveland, OH). In some experiments, 5 μM colchicine (Sigma-Aldrich Inc.) was applied to both the control and experimental groups 2 hours before the addition of A2.

### Electrophysiological Recordings

After the 2 hour A2 incubation time, patch clamp studies of Control and Experimental cells were begun in parallel. Most (85%) of the data were collected within the initial 6 hours of experimentation. On one occasion we continued experiments for 11 hours and could still find some healthy cells, though most were contracted and dying. Cells stored overnight were not usable the next day. Whole-cell patch clamp experiments were conducted at room temperature (22°C). For both K^+^-current and AP measurements, the internal pipette solution contained (in mM) 115 K-Aspartic Acid, 25 KOH, 10 KCl, 3 MgCl_2_, 11 EGTA, 10 HEPES and 5 Na_2_-ATP (pH adjusted to 7.2 with KOH). Pipette series resistances were 4–7 MΩ in whole-cell mode. For K^+^-current measurements, myocytes were perfused with an external solution containing (in mM) 137.7 NaCl, 2.3 NaOH, 5.4 KCl, 1 MgCl_2_, 1.8 CaCl_2_, 2 CoCl_2_, 10 Glucose and 5 HEPES (pH adjusted to 7.4 with NaOH). The Co^2+^ was added to block L-Type Ca^2+^ channels, which activate in the same voltage range as I_to_. For action potential (AP) recordings, Co^2+^ was omitted from the external solution. Surface charge effects due to the divalent Co^2+^ will slightly shift the voltage dependence of the K^+^-currents. However, we assume it did not alter the effects of A2, since Co^2+^ was present in both Experimental and Control solutions.

Both voltage-clamp and current-clamp experiments were performed using an Axopatch 1D amplifier (Axon Instruments Inc.) interfaced to a computer with a Digidata 1200 digitizer and pClamp 8.2 software (Axon Instruments Inc.). APs were elicited in current-clamp mode with short depolarizing current injections (~2 ms) at a frequency of 2.5 Hz (lower stimulation frequencies did not significantly alter the shape of the AP). In voltage-clamp mode, K^+^ currents were measured in response to a short prepulse (10 ms) to -30 mV to inactivate Na^+^ channels followed by 6000 ms voltage steps (V_test_) between +10 mV and +50 mV from a holding potential of -65 mV. For all voltage-clamp recordings, series resistances were compensated electronically by ~85%.

Peak current amplitudes were normalized to the cell capacitance (C_m_) and presented as current densities (pA/pF). Capacitance was measured in each cell using the Membrane Test function in the pClamp software. Average (mean ± S.D.) C_m_ values were 147 ± 24 pF (n = 16) in Control vs 136 ± 16 pF (n = 16) in 5 μM A2. These values were not statistically different based on the Student’s *t-test* (P = 0.15). Unless otherwise stated, pooled data are presented as mean ± SE.

Given a typical pipette resistance of 5 MΩ, which was electronically compensated to give a series resistance of R_S_ = 0.8 MΩ, and an average C_m_ = 140 pF, the recording system had a time constant of R_S_C_m_ = 112 μs. Since the fastest time recorded, τ_fast_ was around 80 ms, there would have been negligible (~0.1%) time distortion due to the recording system. However, the peak K^+^-current density in the mouse myocytes was high relative to other species, and at +50 mV it was about 50 pA/pF, so with C_m_ = 140 pF and R_S_ = 0.8 MΩ, the max voltage drop due to series resistance was about 5 mV. In the presence of A2 the peak currents were reduced to about 60% of control, so the maximum voltage clamp error was around 3 mV. The effects of this error on our conclusions are addressed in **Discussion**.

### Data Analysis and Statistics

Voltage- and current-clamp data were analyzed using Clampfit 8.2 (Axon Instruments Inc.), Microsoft Excel (Microsoft Inc.), SigmaPlot (Systat Software Inc.) and MATLAB (Mathworks Inc.). Action potential durations to 50% and 90% repolarization (APD_50_ and APD_90_, respectively) were determined using MATLAB. Distinct K^+^ current components (I_to,fast_, I_K,slow_ and I_sus_) were extracted from the K^+^-current recordings using the Curve Fitting Toolbox in MATLAB to fit a two-exponential decay function in the form of A_1_exp(-t/τ_1_) + A_2_exp(-t/τ_2_) + A_3_ [29]. A_1_ and A_2_ represent peak amplitudes of I_to,fast_ and I_K,slow_, which have inactivation time constants that differ by a factor of approximately 20. I_to,fast_ was identified as the inactivating component with a decay time constant less than 100 ms and I_K,slow_ was identified as the slower inactivating component (τ_decay_ > 1000 ms). A_3_ represents the steady-state component I_sus_. Percentage inhibitions in I_to,fast_ and I_K,slow_ were plotted as functions of A2 concentration and standard binding curves were fitted to the data using SigmaPlot.

The electrophysiological results had considerable variability, owing to several factors. Relative to myocytes isolated from dog or guinea pig hearts, the isolated mouse myocytes are smaller, do not survive as long, are more difficult to patch clamp, and the patch cannot be held as long. Moreover, mouse myocytes express at least two components of time-dependent K^+^-currents with overlapping voltage-dependencies, so curve fitting is needed to estimate A2 effects on each. Lastly, because of the thinness of the ventricular wall, we could not reliably separate endocardial and epicardial myocytes, so the data are from cells at random locations across the wall. If there is a transmural gradient in endogenous A2, as was found in dog ventricle [4], this would add to the scatter in control responses as well as in the effect of A2. To accommodate these limitations, we executed many experiments and looked for trends in the average responses.

Statistical differences between action potential durations in Control vs. Experimental conditions were assessed with the Student’s *t*-test. Statistical differences between Control and 5 μM A2 current-voltage relationships were assessed with Two Way Repeated Measures Analysis of Variance (ANOVA one factor repetition), with *post hoc* Multiple Comparison Procedures (Holm-Sidak method). The *post hoc* comparison is essentially a conditional probability: given the two graphs differ, does each corresponding pair of experimental points significantly differ.

The relationship between increasing concentrations of A2 and Experimental parameters were tested using Linear Regression Analyses (IBM SPSS Statistics, Version 22). Log A2 concentrations were plotted against experimental results, with a null hypothesis that the slope of the least-squares regression line for the population is zero. The probability that the calculated regression line could be explained by the null hypothesis is measured using the t-statistic. This is equivalent to the null hypothesis that the population correlation coefficient is zero.

## Results

### A2 Dose Dependent Action Potential (AP) Prolongation

**Fig 1:**
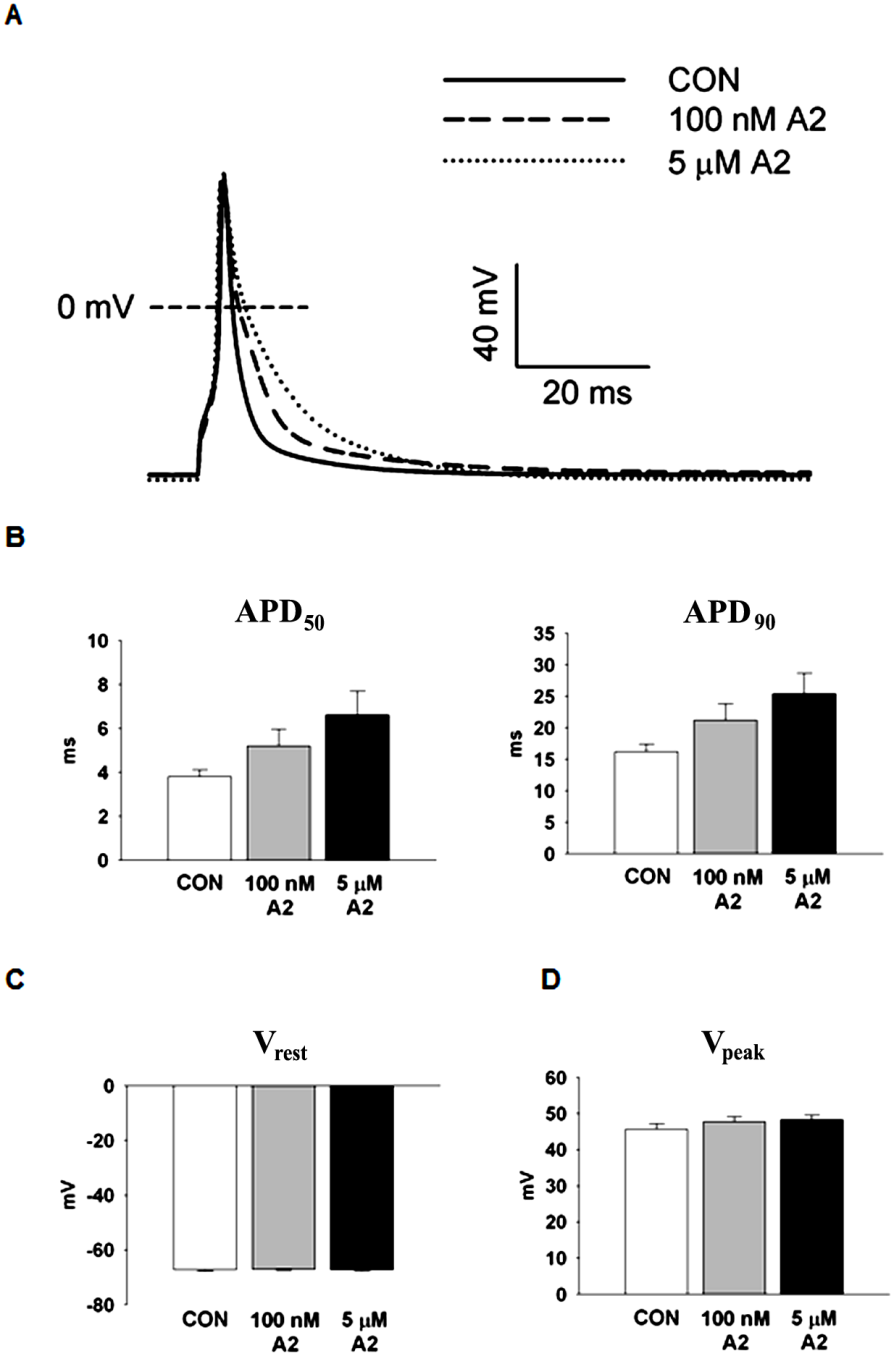
Dose dependence of A2 induced AP prolongation. **(A)** Representative AP waveforms measured from LV myocytes incubated in the presence of zero, 100 nM and 5 μM A2. **(B)** APs display A2 dose-dependent prolongation of early (APD_50_) and late (APD_90_) repolarization. Based on a Regression Analysis, increasing concentrations of A2 caused increases in APDs (P = 0.015). **(C)** A2 stimulated cells exhibit no significant changes in resting potential, V_rest_. **(D)** Peak AP voltage, V_peak_, is also not significantly affected by A2.

Fig 1A presents representative AP recordings from control (CON) myocytes and from myocytes incubated with A2. The myocytes displayed A2 concentration-dependent prolongation in AP duration. Early and late phase repolarizations (APD_50_ and APD_90_, respectively) increase with an increased dose of externally applied A2 (Fig 1B). APD_50_ values were 3.82 ± 0.30 ms (CON, n = 20), 5.19 ± 0.76 ms (100 nM A2, n = 19) and 6.61 ± 1.08 ms (5 μM A2, n = 22). A similar trend in APD_90_ was observed as well: 16.21 ± 1.17 ms (CON), 21.18 ± 2.63 ms (100 nM A2) and 25.37 ± 3.28 ms (5 μM A2). A2 caused no changes in either the resting potential, V_rest_ (Fig 1C) or the peak potential, V_peak_ (Fig 1D). The data indicate A2 stimulation in LV myocytes triggers the remodeling of one or more membrane currents that significantly contribute to action potential repolarization.

### The Voltage Dependence of I_to,fast_ and I_K,slow_ in A2 Stimulated Cells

**Fig 2:**
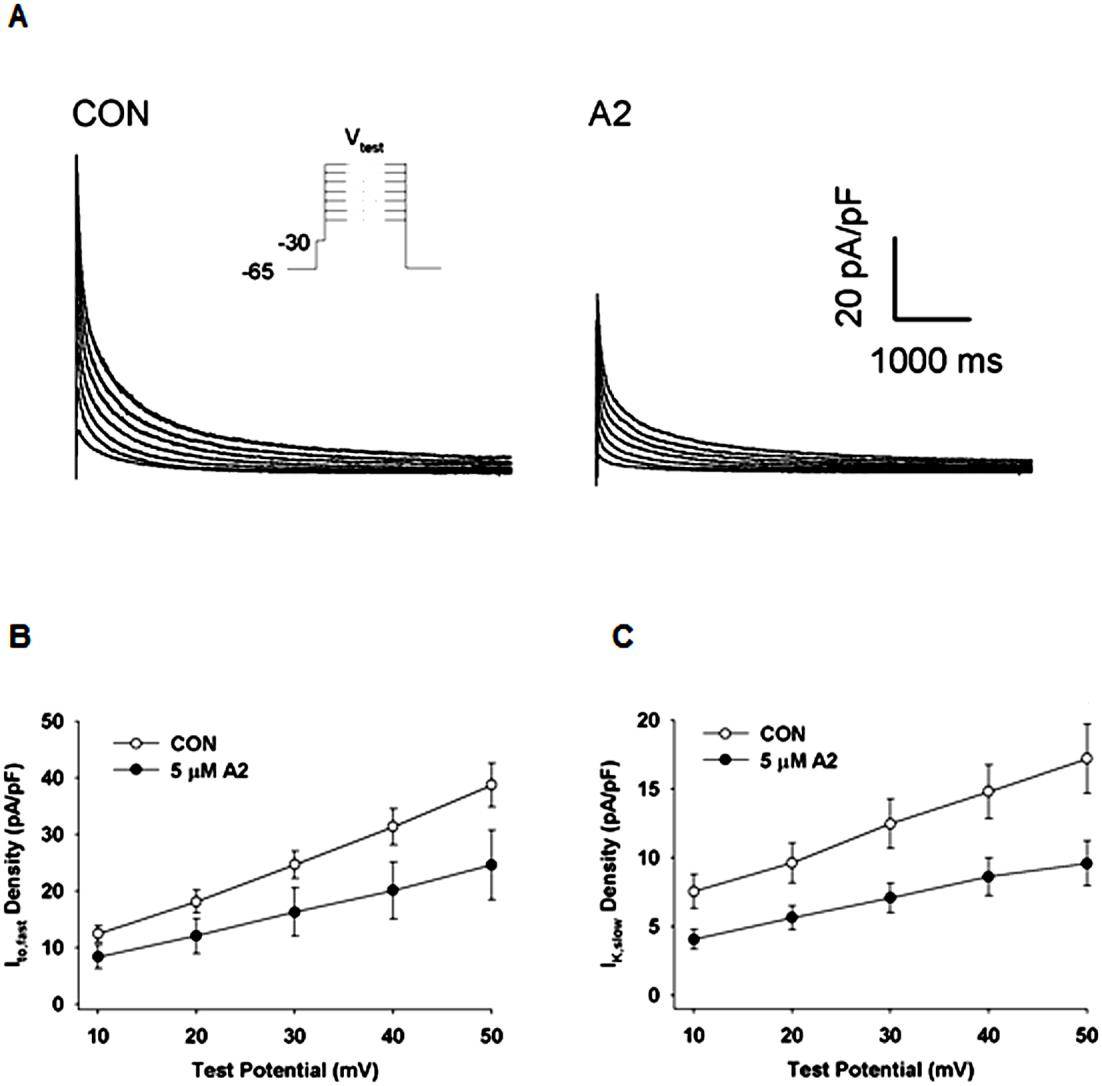
A2 induced reductions in I_to,fast_ and I_K,slow_. **(A)** Representative traces of total outward K^+^-currents recorded from untreated (CON) LV myocytes and those treated with 5 μM. Outward currents were elicited with 6 s test potentials ranging from -10 mV to +50 mV from a holding potential of -65 mV with a 10 ms prepulse to -30 mV to inactivate the fast Na^+^ current, as shown in the inset. Voltage dependences of peak I_to,fast_ **(B)** and I_K,slow_ **(C)** densities were determined by curve fitting two exponentials to the recorded current traces. Based on ANOVA statistical analyses, the overall responses differ significantly (I_to,fast_ P = 0.005, and I_K,slow_ P = 0.001). *Post hoc* statistical analyses of A2 induced changes at each voltage were all significant (P < 0.05).

Voltage clamp recordings obtained from LV cells incubated with 5 μM A2 were compared with recordings measured in CON cells. Fig 2A presents representative traces of the total outward current in CON and 5 μM A2 cells. The voltage clamp protocol showed in the inset to Fig 2A caused a rapid, transient increase in the outward current that was consistent with K^+^ currents that have been described previously in mouse left ventricular myocytes [29]. I_to,fast_ and I_K,slow_ were separated from these traces by fitting exponential functions to the data as described in **Materials and Methods**. Peak current densities at each test potential were averaged and plotted as a function of the test potential (V_test_). Peak I_to,fast_ and I_K,slow_ current-voltage relationships obtained from CON vs 5 μM A2 cells are presented in Fig 2B and 2C.

**Fig 3:**
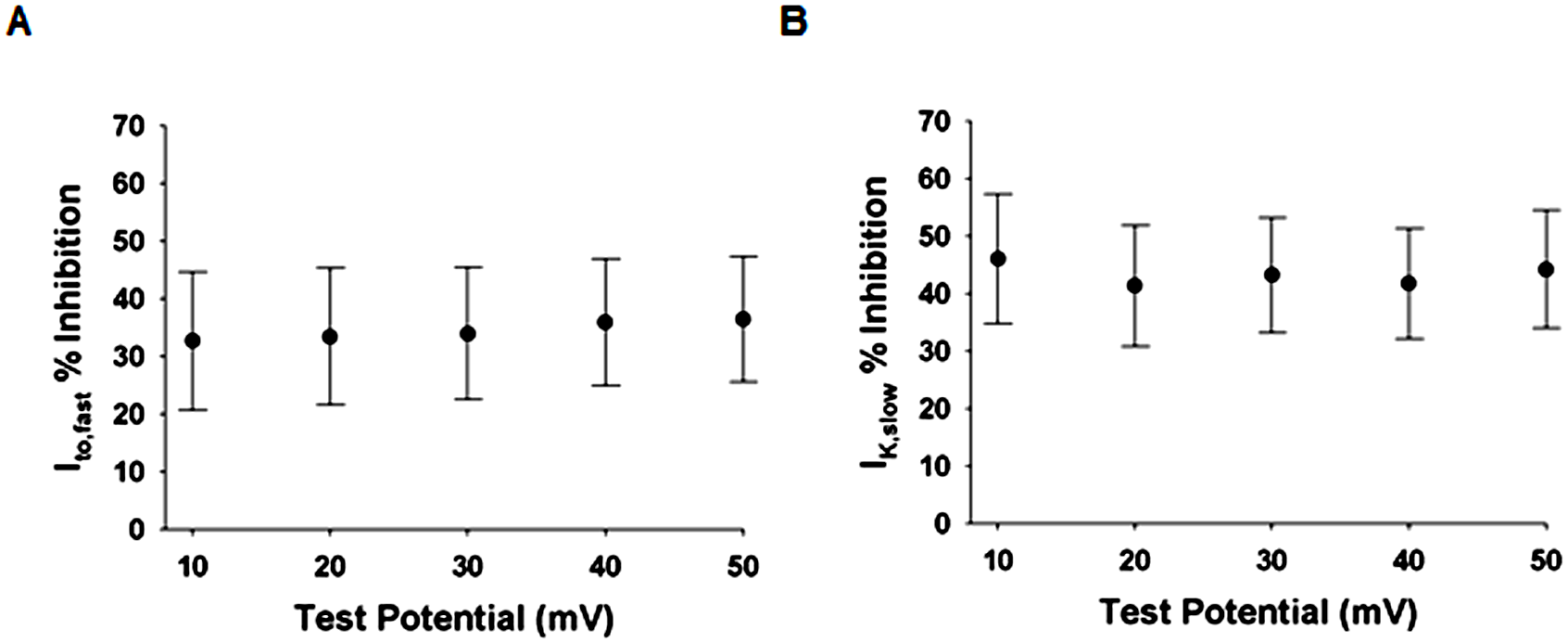
The voltage dependence of A2-induced reductions in peak I_to,fast_ and I_K,slow_. Inhibitions were determined from the voltage dependence of peak I_to,fast_ and I_K,slow_ in control cells (n = 8) and in cells exposed to 5 μM A2 (n = 8). Percent inhibition was calculated from the reduction in current divided by the control current. The percent reductions in I_to,fast_ **(A)** and I_K,slow_ **(B)** exhibit minimal voltage dependence, suggesting A2 reduces the number of open K^+^-channels per unit area of plasma membrane.

A2 effects on the voltage dependences of I_to,fast_ and I_K,slow_ were determined from peak current amplitudes at each test potential measured in CON cells and cells treated with 5 μM A2. A2 induced percent inhibitions of I_to,fast_ and I_K,slow_ were calculated by taking the difference in the mean current amplitude at every test potential between CON and A2 groups and dividing the difference by the mean of the CON group. A2 induced percent inhibitions at test potentials between +10 and +50 mV are shown in Fig 3A (I_to,fast_) and 3B (I_K,slow_). On average, peak currents in A2 stimulated cells were inhibited by 35 ± 11% and 43 ± 10% for I_to,fast_ and I_K,slow_, respectively, with minimal deviations at each test potential. These results suggest that A2 induces reductions in the number of open K^+^ channels per unit area of cell membrane.

### Coherent Regulation of I_to,fast_ and I_K,slow_ in A2 Stimulated Cells

Table 1 displays peak current densities and their inactivation time constants in cells incubated with varying [A2]. Currents were measured at a test potential of +50 mV. Both I_to,fast_ and I_K,slow_ significantly decreased with increasing concentrations of A2. There was also a small but statistically significant increase (about 17%) in τ_slow_ with increasing A2, whereas there was no effect of A2 on τ_fast_. Possible reasons for the apparent increase in τ_slow_ are addressed in **Discussion**.

**Table 1:** A2 dose dependent modulation of K^+^-currents. Regression analyses showed I_to,fast_ and I_K,slow_ significantly decreased with increasing A2 (P < 0.001). τ_slow_ vs. A2 significantly increased with increasing A2 (P < 0.001). The slope of τ_fast_ vs. A2 was not significantly different from zero.

A2 (nM)	0	50	100	200	300	1,000	2,000	5,000	10,000
I_to,fast_ (pA/pF)	37±2	31±5	38±3	29±7	32±4	22±5	23±3	25±6	23±6
τ_fast_ (ms)	76±3	87±5	70±6	60±2	87±5	70±6	81±2	69±5	79±6
I_K,slow_ (pA/pF)	16±1	15±2	16±3	15±3	16±3	11±2	8±1	10±2	8±1
τ_slow_ (s)	1.34 ±.07	1.46 ±.04	1.32 ±.04	1.33 ±.07	1.53 ±.08	1.46 ±.10	1.60 ±.04	1.47 ±.08	1.75 ±.10
n	26	5	8	6	5	6	5	8	7

**Fig 4:**
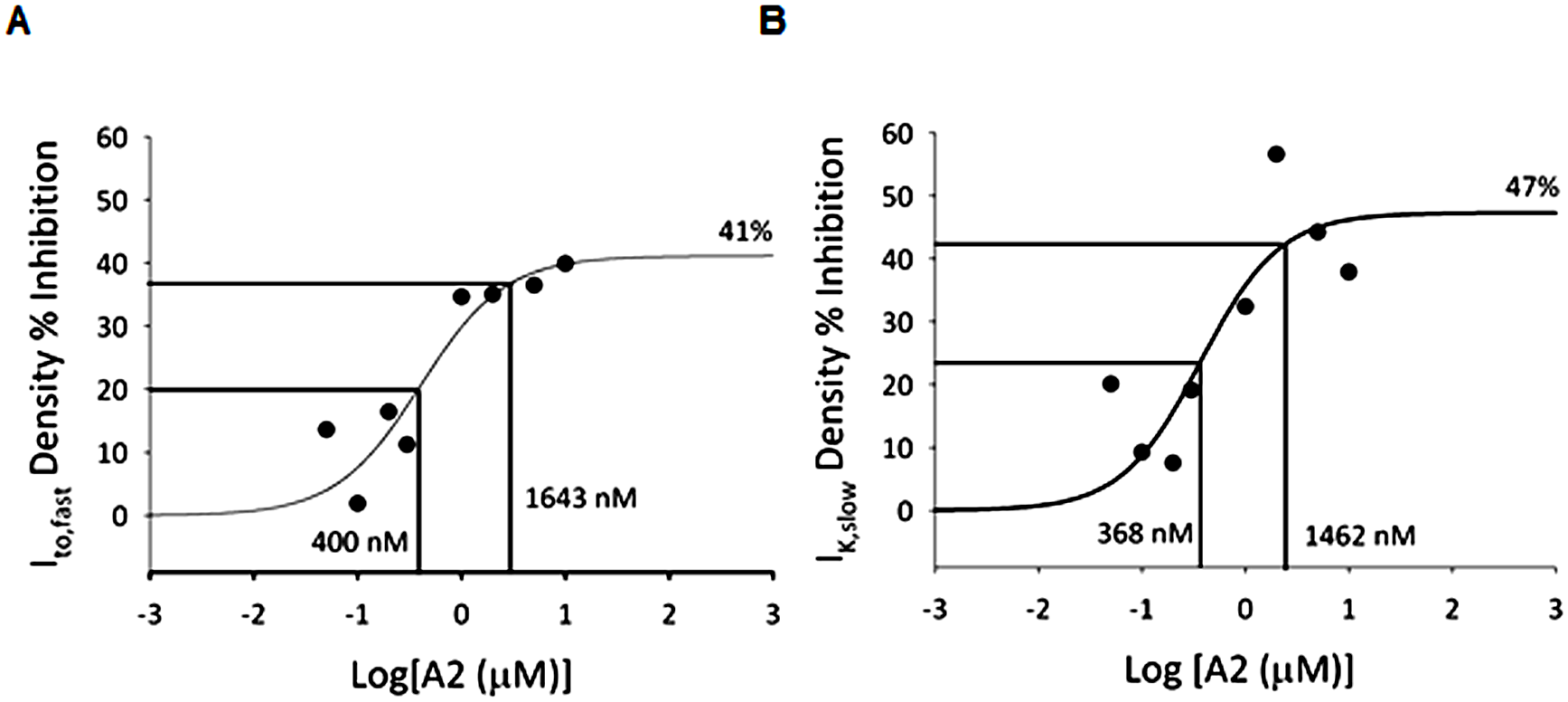
A2-mediated dose-inhibition relationships for I_to,fast_ and I_K,slow_. Percent inhibitions induced by various A2 concentrations (0.05 to 10 μM) were calculated from peak I_to,fast_ and I_K,slow_ values obtained at V_test_ = +50 mV (Table 1). Percent inhibition vs. [A2] relationships of I_to,fast_ **(A)** and I_K,slow_ **(B)** were plotted and standard binding curves were fit to the data. Based on the curve fits, half-maximal reductions of I_to,fast_ and I_K,slow_ correspond to A2 concentrations of 400 nM and 368 nM respectively, and 90%-maximal reductions correspond to 1643 nM and 1462 nM respectively, as indicated in the graphs. Based on the analysis, stimulation with high [A2] can maximally inhibit I_to,fast_ by 41% and I_K,slow_ by 47%.

Relative changes (%) in peak I_to,fast_ and I_K,slow_ at V_test_ = +50 mV were plotted as a function of [A2], and sigmoidal dose-inhibition curves were fit to the data as shown in Fig 4. The dose-inhibition relationships imply that in cells stimulated with saturating [A2], peak I_to,fast_ and I_K,slow_ densities are attenuated by 41% and 47%, respectively. Additionally, the half maximal inhibitory concentrations (K_0.5_) of A2 for I_to,fast_ (400 nM) and I_K,slow_ (368 nM) are similar and may be identical given experimental variability. Likewise, the 90%-maximal inhibitory concentrations (IC_90_) were also similar (I_to,fast_: 1600 nM, I_K,slow_: 1500 nM). These findings suggest A2 stimulation coherently attenuates I_to,fast_ and I_K,slow_ in a dose dependent manner. Thus the mechanisms underlying regulation of these two currents are similar, if not the same.

### Colchicine Inhibition of AT_1_R Mediated Electrical Remodeling

**Fig 5:**
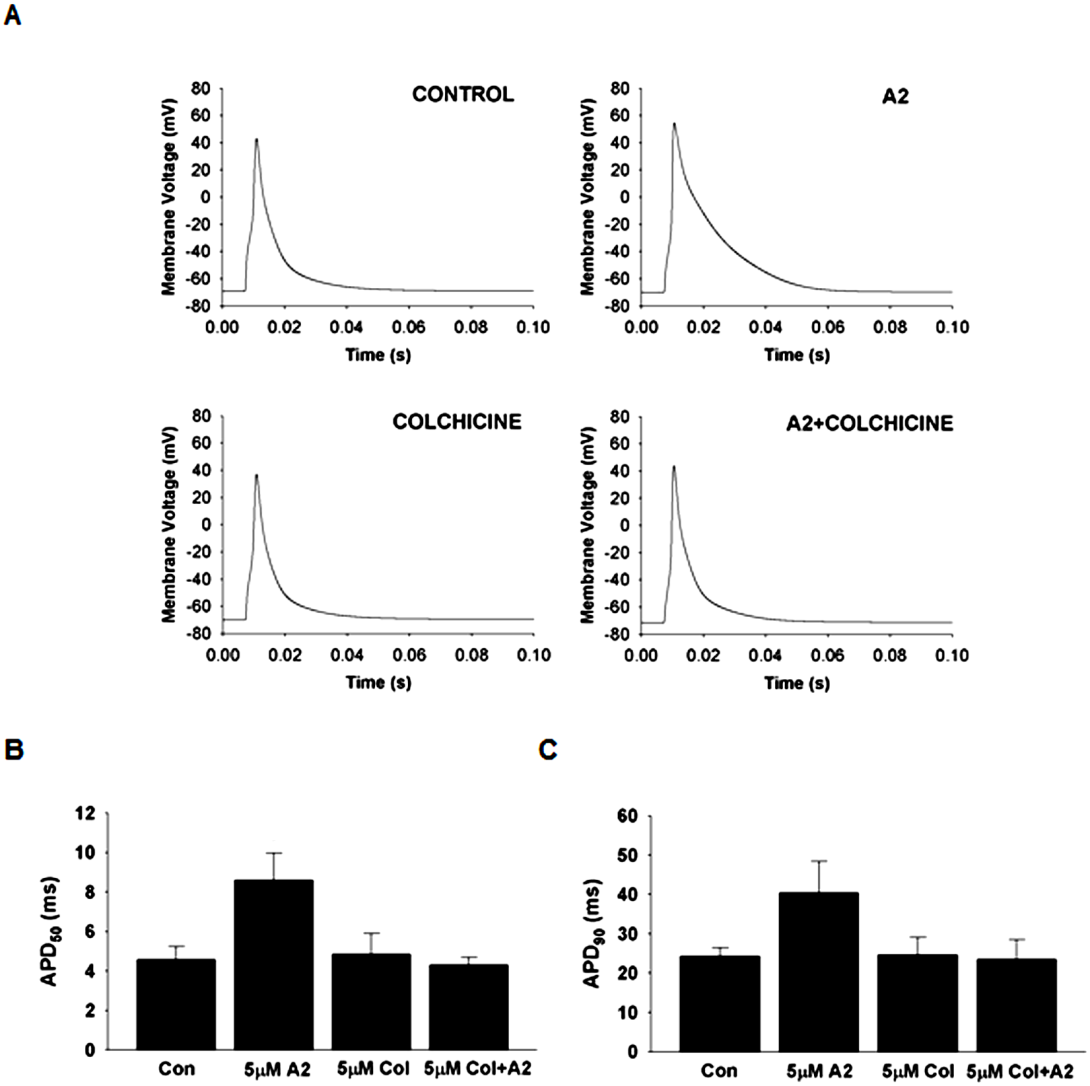
A2-induced electrical remodeling was blocked in cells pretreated with colchicine for 2 hours prior to A2 treatment. **(A)** Example traces of action potentials recorded from untreated, 5 μM colchicine treated, 5 μM A2 treated, and both colchicine+A2 treated cells. Repolarization delays were observed in A2 treated myocytes as indicated by prolonged APD_50_ **(B)** and APD_90_ **(C)** but these effects were blocked in cells pretreated with colchicine. These results suggest that A2 induced electrical remodeling requires functioning intracellular transport mechanisms and may involve the internalization of AT_1_Rs, ion channels or both. Based on the students *t-test*, A2 significantly increased the APD_50_ (P < 0.05) but the increase in APD_90_ did not quite reach the standard for significance (P = 0.06). However, given these results and the data in Fig 1, it seems reasonable to suggest that A2 increased AP duration in control, but there is no A2 effect when the cells were pretreated with colchicine.

Colchicine disrupts vesicular transport by binding to tubulin and inhibiting microtubule polymerization. Fig 5 presents the effect of colchicine on A2-induced electrical remodeling in mouse myocytes. Fig 5A shows representative APs from untreated (Control), colchicine treated, A2 treated, and both colchicine and A2 treated cells. Fig 5B & 5C show APD_50_ and APD_90_ measured from the same four groups of cells. APDs in CON cells were significantly shorter than those in A2 treated cells (CON APD_50_: 4.59 ± 0.64 ms and APD_90_: 24.33 ± 2.15 ms, n = 6), and (A2 treated cells APD_50_: 8.61 ± 1.37 ms and APD_90_: 40.40 ± 7.95 ms, n = 6). Whereas no significant difference was observed between CON and Col treated cells (Col APD_50_: 4.85 ± 1.05 ms and APD_90_: 24.65 ± 4.39 ms, n = 6) or between CON and Col+A2 treated cells (Col+A2 treated APD_50_: 4.29 ± 0.40 ms and APD_90_: 23.43 ± 5.04, n = 6). Though colchicine alone appears to have no effect on the AP, pre-incubation with colchicine blocks A2 induced changes in AP morphology. Thus inhibition of microtubule assembly and disruption of vesicular trafficking alters the normal activity of AT_1_Rs in mouse and dog heart [30], possibly by blocking receptor internalization. Another possibility is that I_to,fast_ and I_K,slow_ internalize upon A2 stimulation under normal conditions and disruption of the microtubule network prevents their internalization. Either possibility is consistent with our data, or perhaps both if receptor and channels are co-internalized upon receptor activation.

### AT_1_R Mediated Electrical Remodeling is via AT_1_R Internalization

**Fig 6:**
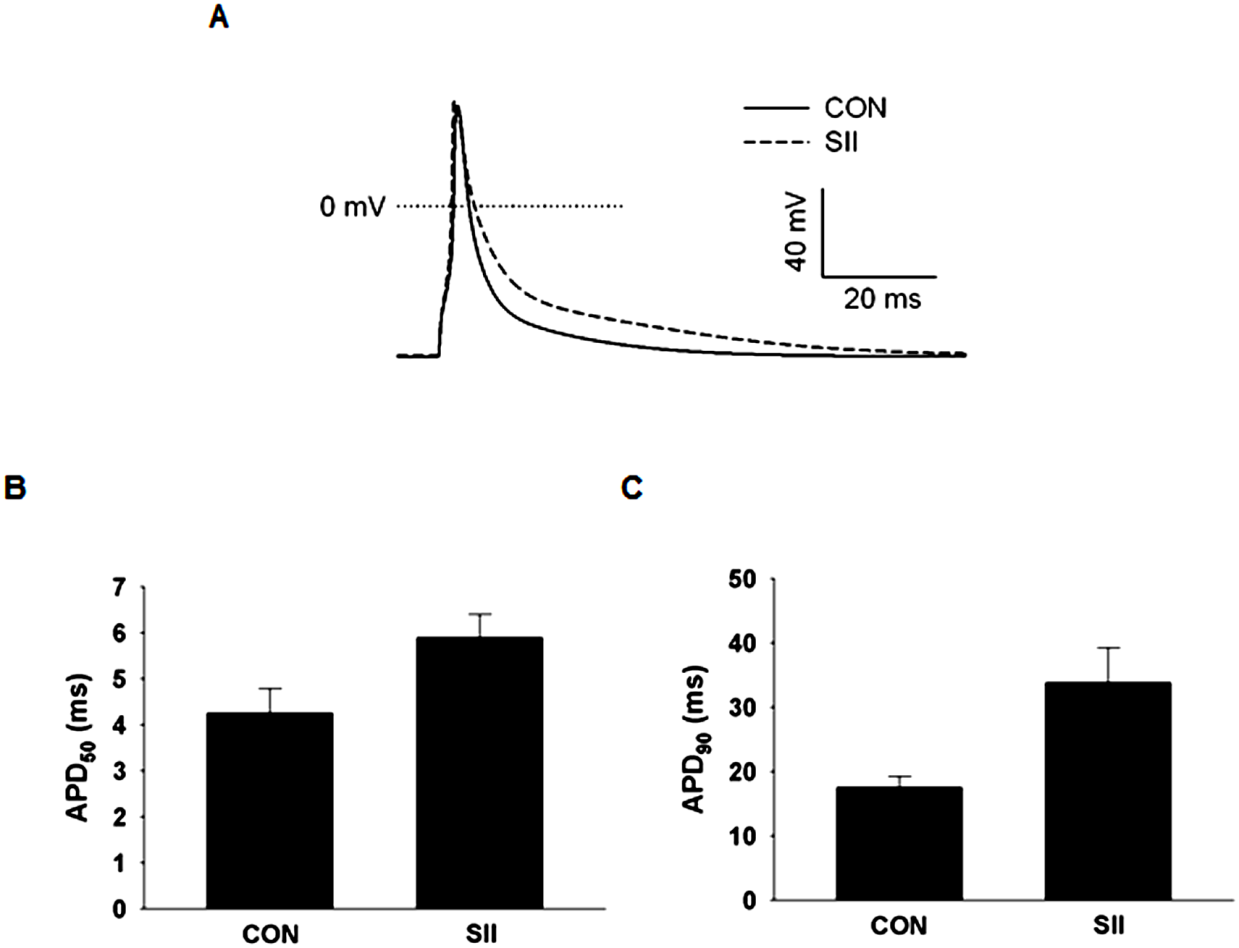
AT_1_R-mediated effects on AP duration do not involve the initial G-protein stimulation that follows receptor activation. **(A)** Representative traces of AP morphology in CON and 5 μM SII incubated cells. SII induced increases in APD, which appears to occur through a G-protein independent β-arrestin2 mediated pathway involving AT_1_R internalization. APDs measured from SII treated cells display significant prolongations in both APD_50_ **(B)** and APD_90_ **(C)**. Based on the student’s *t-test*, APDs at both levels of repolarization are significantly increased (P < 0.05).

Cells were incubated with 5 μM SII ([Sar^1^-Ile^4^-Ile^8^]A2), which selectively activates β-arrestin2 dependent signaling and receptor internalization without G protein stimulation [13,14]. APs were recorded from CON and SII treated cells (Fig 6). APs from SII treated cells were significantly prolonged compared with controls (CON 4.26 ± 0.53 ms and SII 5.89 ± 0.51 ms for APD_50_; CON 17.55 ± 1.70 ms and SII 33.81 ± 5.39 ms for APD_90_), similar to the prolongations observed in cells treated with A2 (Fig 1). These results suggest electrical remodeling occurs through β-arrestin2 dependent AT_1_R internalization without the involvement of AT_1_R activated G proteins.

**Fig 7:**
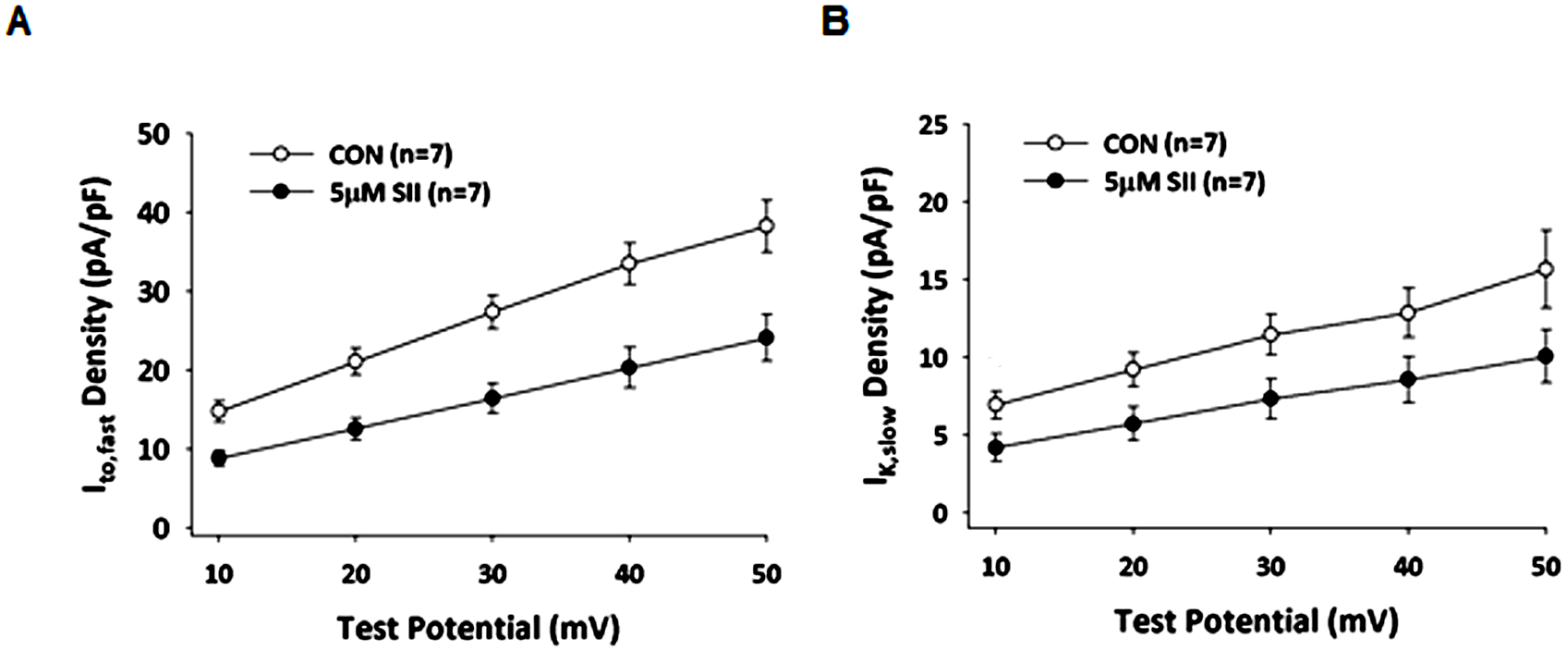
AT_1_R-mediated inhibition of repolarizing K^+^-currents does not involve the initial G-protein stimulation that follows receptor activation. Internalization of AT_1_Rs by incubation in 5 μM SII caused reductions in I_to,fast_ **(A)** and I_K,slow_ **(B)**. Based on ANOVA statistical analyses, the overall responses differ significantly (I_to,fast_ P = 0.004, and I_K,slow_ P = 0.025). *Post hoc* statistical analyses of SII induced changes at each voltage were significant (P < 0.05) with exceptions of the results for I_K,slow_ obtained at 10 mV and 20 mV, which did not reach statistical significance.

Additionally, K^+^ currents were recorded from CON and SII treated cells to examine the effect on peak I_to,fast_ and I_K,slow_ (Fig 7). Compared with CON cells, peak I_to,fast_ (Fig 7A) and I_K,slow_ (Fig 7B) densities were significantly attenuated in SII treated cells (I_to,fast_ 41%, I_K,slow_ 44% inhibition at V_test_ = +50 mV). These inhibitions are essentially the same as A2-mediated inhibitions reported for the data in Fig 2 or Table 1. Thus, consistent with the AP results (Fig 6), the effect of AT_1_R activation on I_to,fast_ and I_K,slow_ appear to be through β-arrestin2 dependent AT_1_R internalization without the involvement of AT_1_R G-protein signaling.

## Discussion

The data presented here are consistent with the presence of a renin-angiotensin-system (RAS) in mouse heart. Though we studied only regulation of transient outward K^+^-currents by the mouse RAS, the results are consistent with the data and hypotheses presented in [4]. These hypotheses were based on data from several transporters, primarily from dog heart. Isolated myocytes from dog hearts are much easier to study electrophysiologically than those from mouse hearts, so the autocrine RAS is more extensively characterized in dog. However the mouse can be genetically modified to provide different insights into this system. Though there are significant differences in the electrophysiology of dog and mouse myocytes, our results suggest they may both express a similar autocrine RAS.

### A2-Mediated Regulation of K-Currents in Mouse Heart

In the mouse heart, the transient K^+^-current decays with fast and slow components, which are thought to be due to independent channels. The proposed molecular relationships are Kv4.2 and Kv4.3 mediate I_to,fast_ [31,32] and Kv1.5 and Kv2.1 mediate I_K,slow_ [29,33]. A2-mediated maximal inhibition of I_to,fast_ was 36–41% whereas maximal inhibition of I_K,slow_ was 41–47%. However, the currents were coherently inhibited by A2 in a dose dependent manner with 50% inhibition at [A2] ≈ 400 nM, suggesting both types of channels may be inhibited by the same mechanism. Inhibition was voltage independent and could be blocked by pretreatment with colchicine, suggesting regulation involved protein trafficking. Although I_to_ proteins in mouse are different than those in dog, there are many parallels between the effects of the autocrine RAS on transient K^+^-currents in these two species.

The lack of any effect of A2 on the voltage dependence of peak current in either dog or mouse myocytes suggests the currents may be reduced by reducing the number of open channels in the plasma membrane. One mechanism to explain this observation is that the channel forming proteins were endocytosed with activated AT_1_Rs to a pool of endosomal vesicles. This hypothesis is also consistent with the colchine result. Moreover, Kv4.3 is one of the proteins involved in I_to,fast_ in mouse. Kv4.3 has been shown in expression systems to co-internalize with activated AT_1_Rs [25]. Our data suggest I_to,fast_ and I_K,slow_ regulation by A2 is through the same mechanism, so co-internalization with the AT_1_R is our working hypothesis.

In mouse heart cells, we used a “biased agonist” that selectively activated β-arrestin2 dependent signals and receptor internalization without G protein stimulation [13, 14]. The “biased agonist” had the same effect on transient K^+^-currents as A2, indicating the initial G protein mediated phase of AT_1_R activation does not contribute to AT_1_R mediated regulation of these currents. Rather, it is the second phase of receptor internalization that inhibits the currents. Hence coupled internalization of I_to_ proteins with AT_1_Rs provides a simple working hypothesis for A2-mediated inhibition of K^+^-currents.

Half maximum inhibition of I_to,fast_ and I_K,slow_ occurred at the same [A2] ≈ 400 μM, so they are most likely regulated by the same mechanism. The 50% inhibition concentrations of A2 in either mouse or dog were orders of magnitude greater than typical plasma concentrations, which are 10–30 pM. Thus the systemic [A2]’s are too low to regulate the cardiac RAS. In dog myocytes, this exceptionally high concentration was shown to occur by autocrine secretion into the T-system lumen, which has an extremely high surface to volume ratio. Hence, low rates of secretion could yield high local concentrations, without disturbing systemic [A2]. The similarly high K_0.5_ in mouse heart cells suggests the presence of a similar system. Moreover, the AT_1_Rs in both mouse and dog heart have a much lower affinity for A2 than receptors in the vasculature, implying cardiac receptors have different post translational modifications, but these remain unknown.

### Uncertainties and Technical Limitations

In **Materials and Methods** we estimated that the time constant for whole cell patch clamp of isolated mouse myocytes was about 112 μs. This is nearly 1000 times faster than the fastest time constants we studied, so there is no significant uncertainty in time resolution. However, the repolarizing current density in mouse myocytes is relatively large, and the sum of peak I_to,fast_ plus I_K,slow_ in control myocytes varied from about 20 pA/pF at +10 mV to 50 pA/pF at +50 mV. This would induce voltage drops across the pipette resistance of about 2 mV to 5 mV. Thus the myocyte membrane voltage actually varied from about +8 mV to +45 mV. In the presence of A2, the current densities varied from about 13 pA/pF to 33 pA/pF so the voltage shifts were from about 1 mV to 3 mV and the membrane voltage actually varied from approximately +9 mV to +47 mV. The data presented here are therefore somewhat voltage shifted from the actual voltage dependence of the repolarizing currents, however the shifts are not large and the estimates are crude so we made no attempt to make corrections. Moreover, these shifts do not alter the conclusion that A2 inhibits the currents.

Based on the linear regression, the time constant τ_slow_ increased from 1.32 s in Control to 1.55 s in 10 μM A2, an increase of 17% (P < 0.001). The increase is small relative to the standard deviations in the data, so even though it shows statistical significance, it should be interpreted with caution. If true, we can think of three possible interpretations of this result: 1) the internalization hypothesis is either not correct or too simple and A2 is also affecting channel gating; 2) this is a systematic artifact arising from the voltage dependence of the time constant and current dependent shifts in the actual membrane voltage; 3) I_K,slow_ comprises two types of channels with slightly different inactivation kinetics, and one type is selectively internalized with the AT_1_R. Any one of these three possibilities is consistent with our data.

### Is A2 Secretion Induced by Strain to Increase Contractility?

There is circumstantial evidence that endogenous A2 is secreted in response to increases in strain and causes electrical remodeling that increases contractility. This hypothesis is based on several studies: Sadoshima et al. [21] showed strain-induced autocrine secretion of A2 in cultured rat cardiac myocytes; Streeter et al. [26] showed a transmural gradient in strain in canine LV; Cordiero et al. [34] showed a transmural gradient in contractility in canine LV; Gao et al. [4] showed a transmural gradient in autocrine secretion of A2, which induced electrical remodeling that increased calcium entry in canine LV. Moreover, effects of A2 stimulation on AP morphology have been documented from other species including guinea pig LV [17] and rabbit LV [19]. Here, we have not studied the mechanism by which endogenous A2 is increased, but we have shown that increasing exogenous A2 inhibits the transient outward K^+^-currents and increases AP duration. In isolated canine myocytes, Dong et al. [35] used the dynamic voltage clamp to specifically subtract I_to_ from the action potential. The result was an increase in AP duration, allowing more time for Ca^2+^ entry through L-type Ca^2+^-channels and increased contractility. We expect the same situation will apply when I_to_ is reduced in mouse myocytes. Thus our data from mouse LV fit with the general picture that is emerging from a number of studies.

The data presented here in connection with other data in the literature suggest an autocrine RAS is a ubiquitous and fundamental property of the heart. Although there are significant differences in APs from dog vs. mouse heart cells, there are significant parallels in the effects of A2 on transient outward K^+^-currents. Our working hypothesis follows that presented in Gao et al.: An autocrine RAS is expressed in cardiac myocytes as a feedback system that responds to increases in strain by secretion of A2. A2 initially causes electrical remodeling, which increases contractility. Long term increases in A2 secretion lead to hypertrophy [21]. While the evidence for this hypothesis is largely circumstantial, the mouse heart provides a venue in which it can be more directly tested.

## Supporting Information

S1 Dataset Original data for Figs 1–7 and Table 1. (XLSX)
